# Supplementary material for: Polycyclic Phenol Derivatives from the Leaves of Spermacoce latifolia and Their Antibacterial and α-Glucosidase Inhibitory Activity
Source: Molecules. 2022 May 22;27(10):3334. doi: 10.3390/molecules27103334 (PMC9145846; doi:10.3390/molecules27103334)

Supporting Information File for molecules-1713636

Polycyclic phenol derivatives from the leaves of *Spermacoce latifolia* and their antibacterial and *α*-glucosidase inhibitory activity.

Shao-Bo Liu [1,†], Lei Zeng [2,†], Qiao-Lin Xu [2,*], Ying-Le Chen [2], Tao Lou [1], Shan-Xuan Zhang [1] and Jian-Wen Tan [1,*]

[1] State Key Laboratory for Conservation and Utilization of Subtropical Agro-bioresources/Guangdong Key Laboratory for Innovative Development and Utilization of Forest Plant Germplasm,

College of Forestry and Landscape Architecture, South China Agricultural University,

Guangzhou 510642, China; sbliu0511@126.com (S.-B.L.); ltao99@yeah.net (T.L.); sxzhang@163.com (S.-X.Z.)

[2] Guangdong Provincial Key Laboratory of Silviculture, Protection and Utilization, Guangdong Academy of Forestry, Guangzhou 510520, China; zenglei@sinogaf.cn (L.Z.); cyingl@sinogaf.cn (Y.-L.C.)

* Correspondence: qlxu@sinogaf.cn (Q.-L.X.); jwtan@scau.edu.cn (J.-W.T.)

† These authors contributed equally to this work.

Table of Contents:

Figure S1: HR-ESI-MS(−) spectrum of compound **1** ……………………………………………….………..….…….……….3

Figure S2: ^{1}H NMR spectrum of compound **1** ………………………………………………………………..…….……4

Figure S3: ^{13}C NMR spectrum of compound **1** …………………………………………………….………………....5

Figure S4: HSQC spectrum of compound **1** ………………………………………………………………...…………6

Figure S5: HMBC spectrum of compound **1** ……………………………………………………………...............7

Figure S6: HR-ESI-MS(−) spectrum of compound **2** ………………………………………………………………8

Figure S7: ^{1}H NMR spectrum of compound **2** ……………………………………………………………………..……9

Figure S8: ^{13}C NMR spectrum of compound **2** …………………………………………………….…………….…………..10

Figure S9: HSQC spectrum of compound **2** ………………………………………………………….……………….…...11

Figure S10: HMBC spectrum of compound **2** ……………………………………………………………….…….………12

Figure S11: HR-ESI-MS(+) spectrum of compound **8** ………………………………………………….…….……...13

Figure S12: ^{1}H NMR spectrum of compound **8** …………………………………………………………….….…………14

Figure S13: ^{13}C NMR spectrum of compound **8** ………………………………………………………….……..………15

Figure S14: HSQC spectrum of compound **8** ……………………………………………………………….….……….…16

Figure S15: HMBC spectrum of compound **8** ………………………………………………………….…….…………17

Figure S1: HR-ESI-MS(−) of compound **1**

Mass Spectrum SmartFormula Report

Analysis Info

Analysis Name	D:\Data\MS\data\202107\tanjianwen_LS-11_neg.d	Acquisition Date	7/22/2021 10:32:46 AM
Method	POS_100-2000_Dirrect Infusion.m	Operator	BDAL@DE
Sample Name	SCSIO	Instrument / Ser#	maXis 29
Comment			

Acquisition Parameter

Source Type	ESI	Ion Polarity	Negative	Set Nebulizer	0.3 Bar		
Focus	Not active	Set Capillary	3500 V	Set Dry Heater	180 °C		
Scan Begin	100 m/z	Set End Plate Offset	-500 V	Set Dry Gas	4.0 l/min		
Scan End	2500 m/z	Set Collision Cell RF	2000.0 Vpp	Set Divert Valve	Waste		

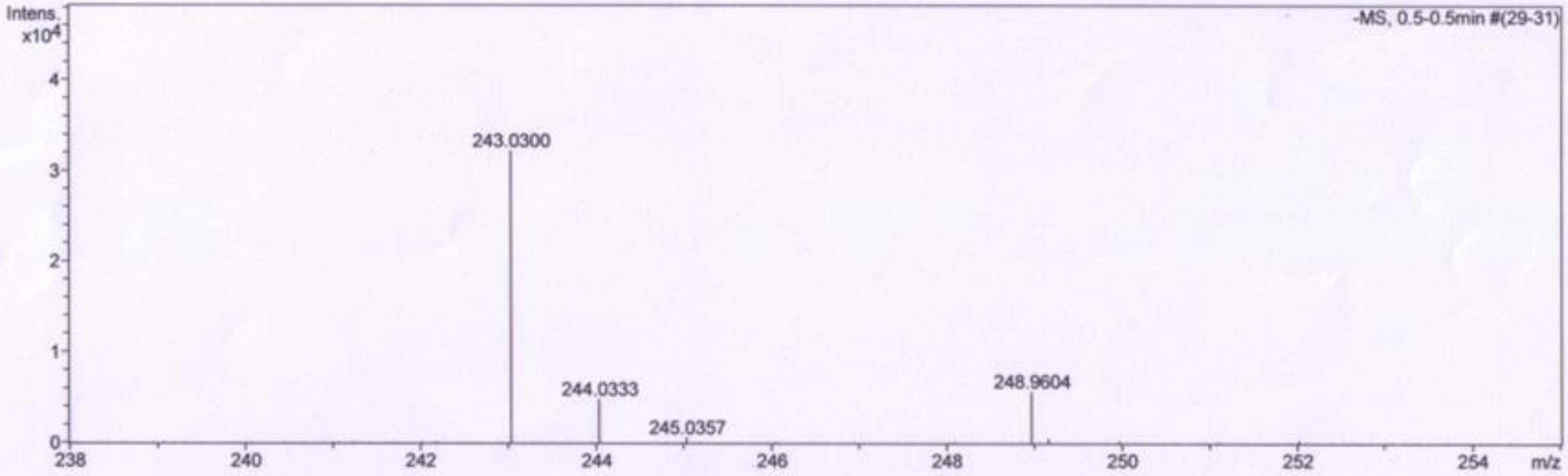

Meas. m/z	#	Formula	m/z	err [ppm]	Mean err [ppm]	rdb	N-Rule	e⁻ Conf	mSigma	Std I	Std Mean m/z	Std I VarNorm	Std m/z Diff	Std Comb Dev
243.0300	1	C 13 H 7 O 5	243.0299	-0.3	-0.4	10.5	ok	even	4.7	8.8	0.2	3.6	0.0	842.7

Figure S2: ^{1}H-NMR spectrum of compound **1**

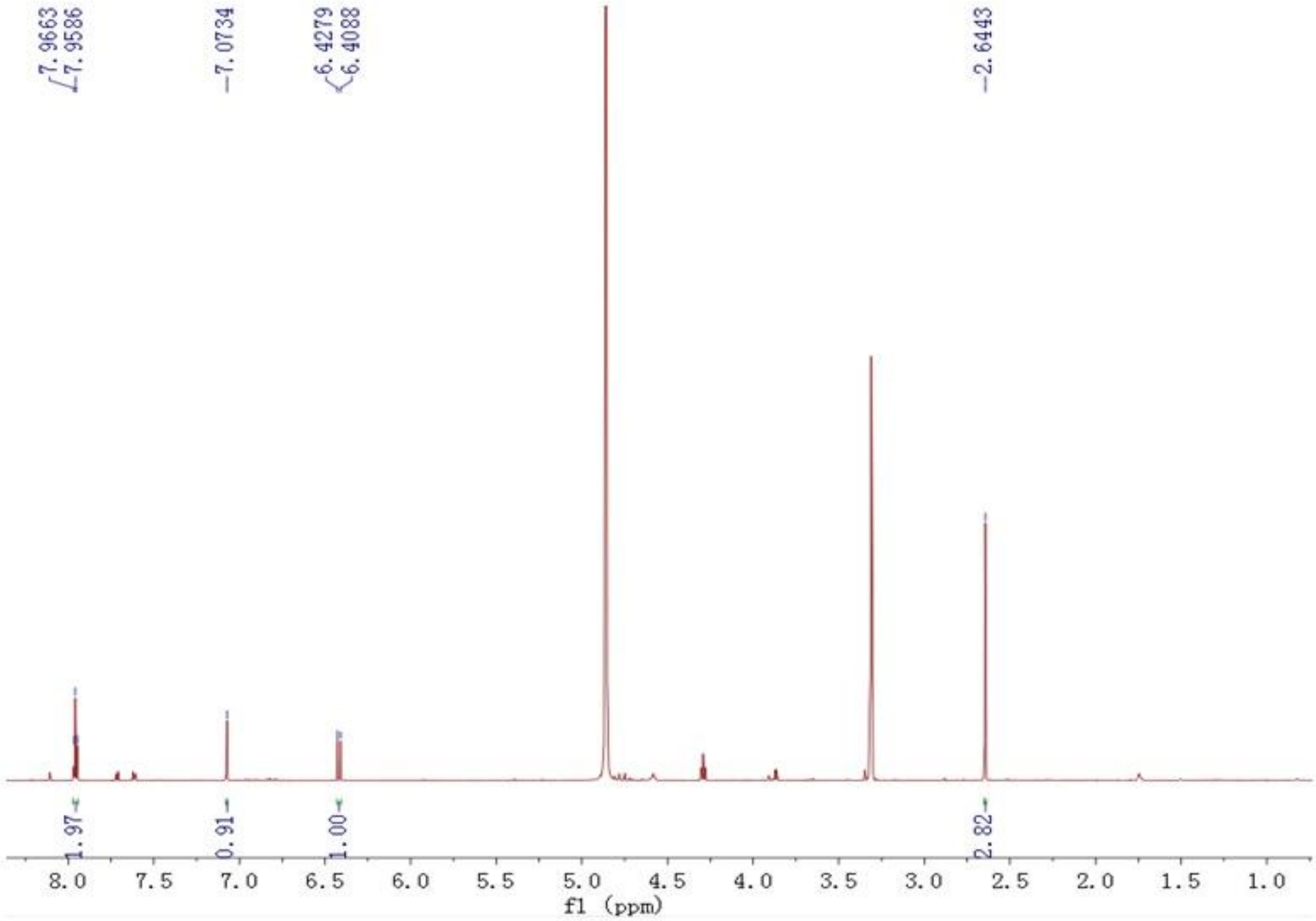

Figure S3: ^{13}C-NMR spectrum of compound **1**

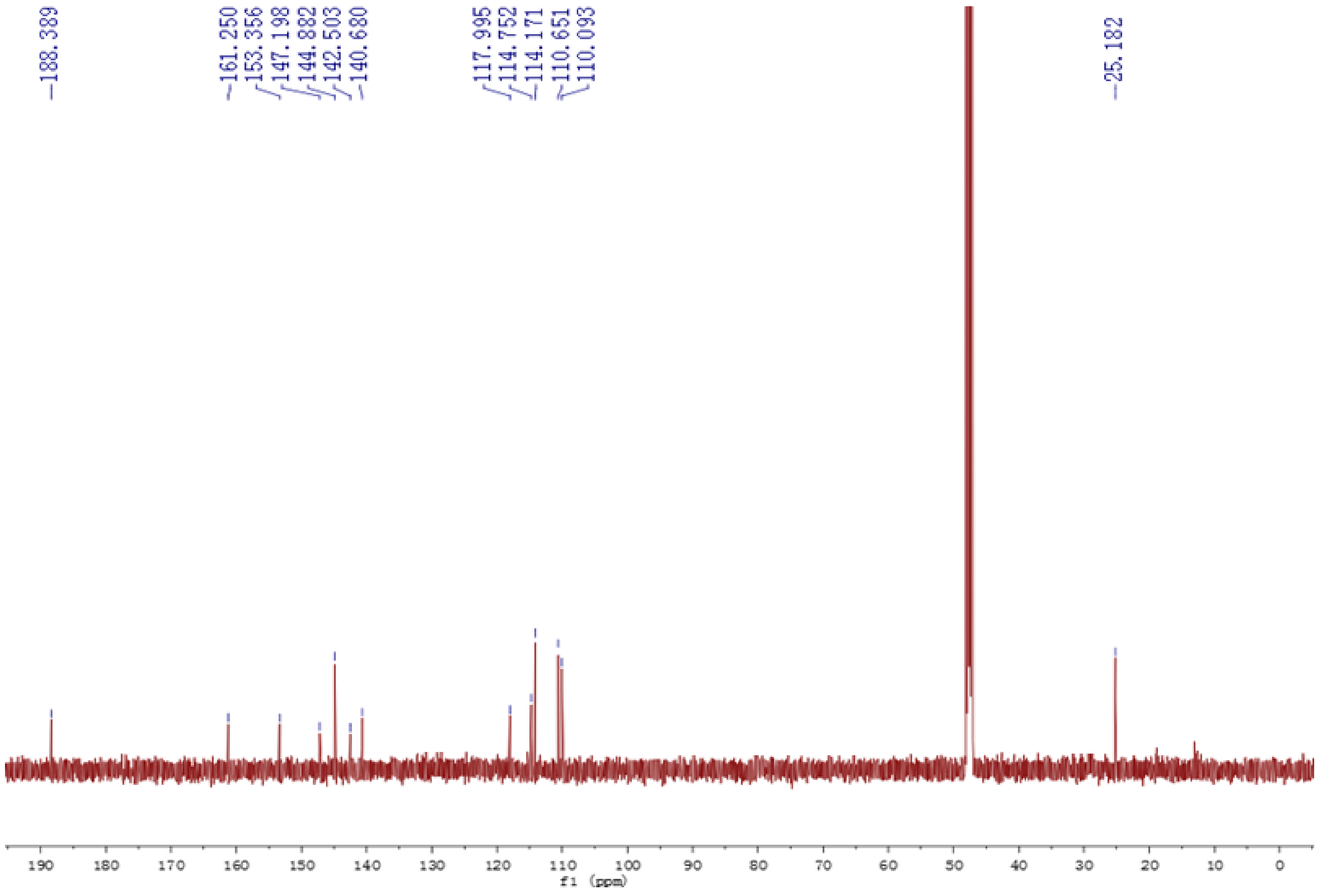

Figure S4: HSQC spectrum of compound **1**

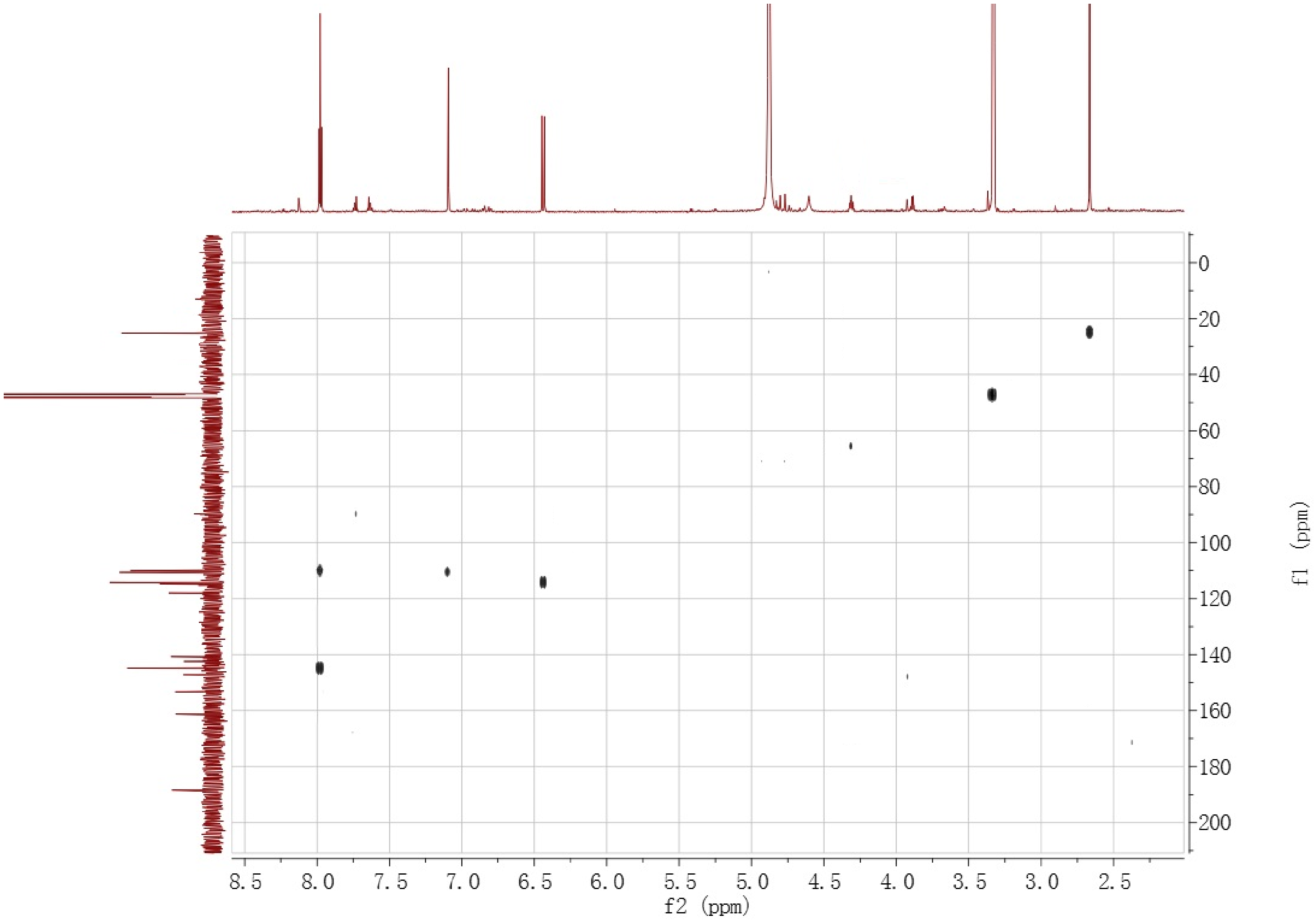

Figure S5: HMBC spectrum of compound **1**

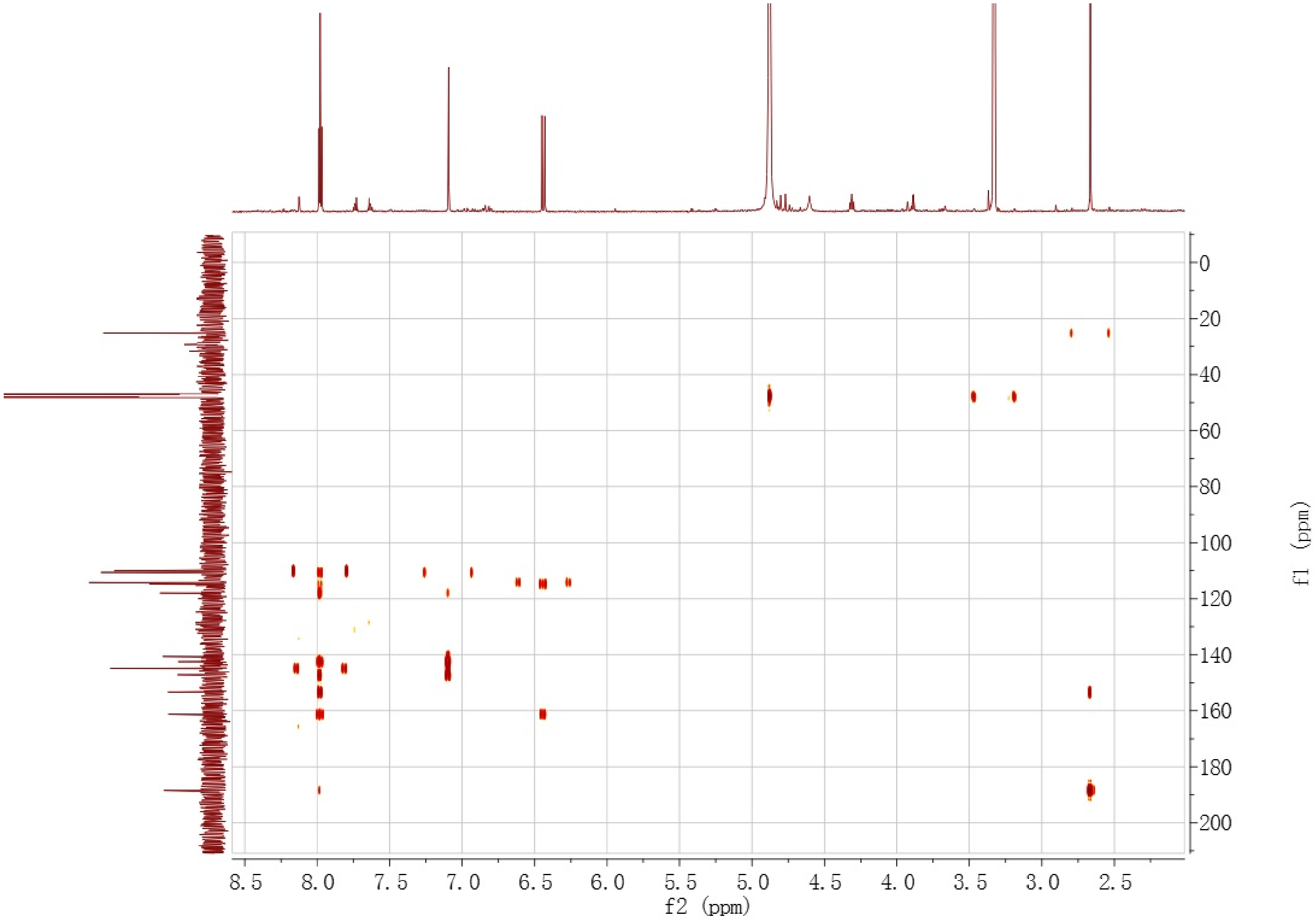

Figure S6: HR-ESI-MS(−) of compound **2**

Mass Spectrum SmartFormula Report

Analysis Info

Analysis Name	D:\Data\MS\data\202107\tanjianwen_LB-47_neg.d	Acquisition Date	7/22/2021 10:27:24 AM
Method	POS_100-2000_Dirrect Infusion.m	Operator	BDAL@DE
Sample Name	SCSIO	Instrument / Ser#	maXis 29
Comment			

Acquisition Parameter

Source Type	ESI	Ion Polarity	Negative	Set Nebulizer	0.3 Bar		
Focus	Not active	Set Capillary	3500 V	Set Dry Heater	180 °C		
Scan Begin	100 m/z	Set End Plate Offset	-500 V	Set Dry Gas	4.0 l/min		
Scan End	2500 m/z	Set Collision Cell RF	2000.0 Vpp	Set Divert Valve	Waste		

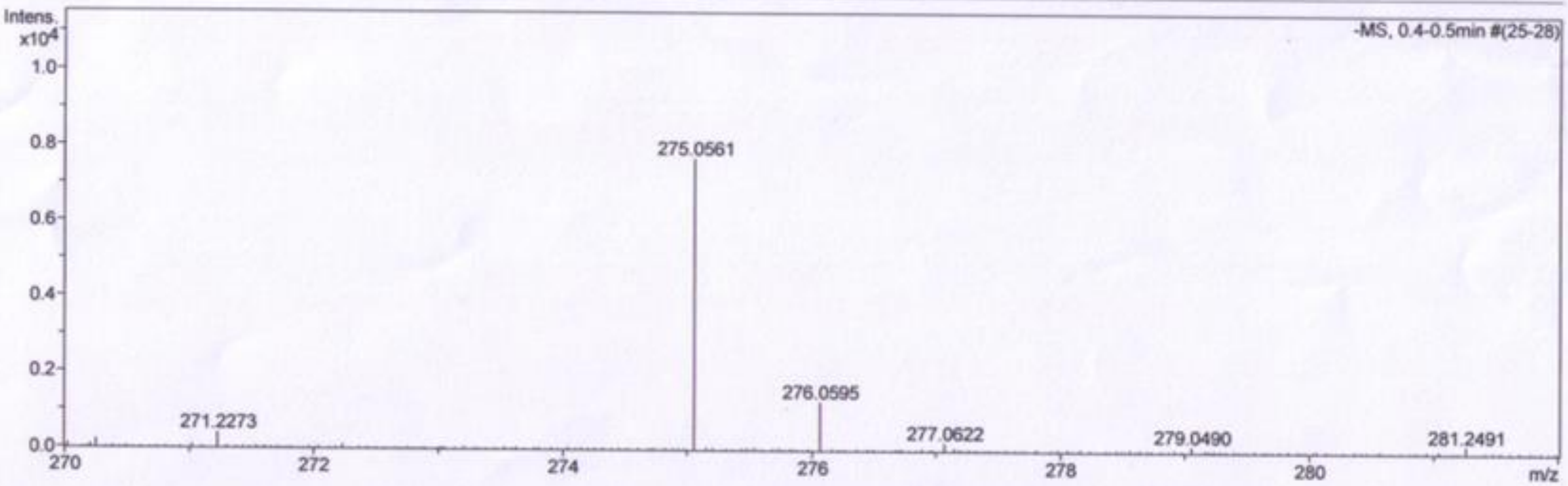

Meas. m/z	#	Formula	m/z	err [ppm]	Mean err [ppm]	rdb	N-Rule	e⁻ Conf	mSigma	Std I	Std Mean m/z	Std I VarNorm	Std m/z Diff	Std Comb Dev
275.0561	1	C 14 H 11 O 6	275.0561	0.2	0.0	9.5	ok	even	7.5	14.9	0.3	6.2	0.0	842.7

Figure S7: ^{1}H-NMR spectrum of compound **2**

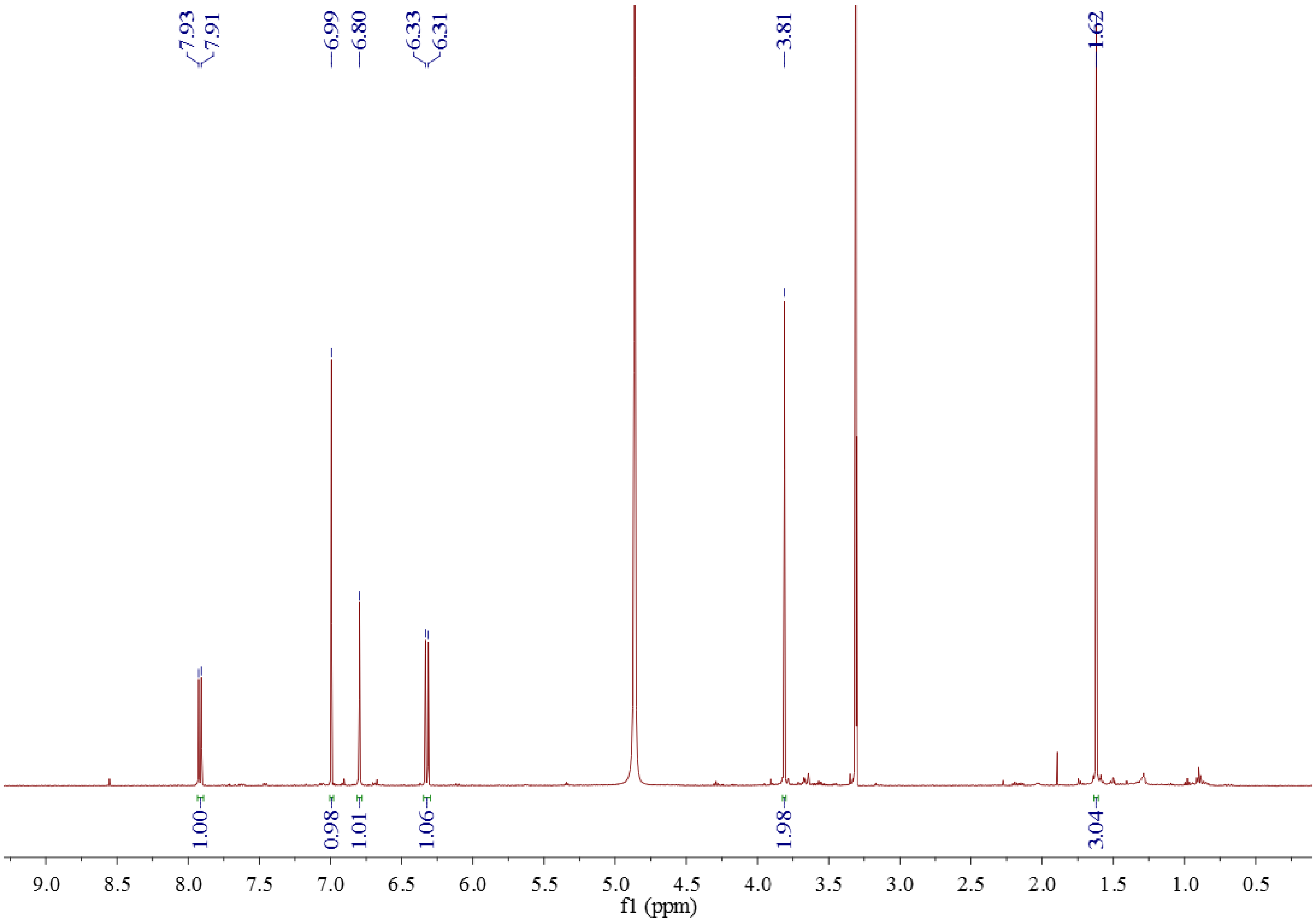

Figure S8: ^{13}C-NMR spectrum of compound **2**

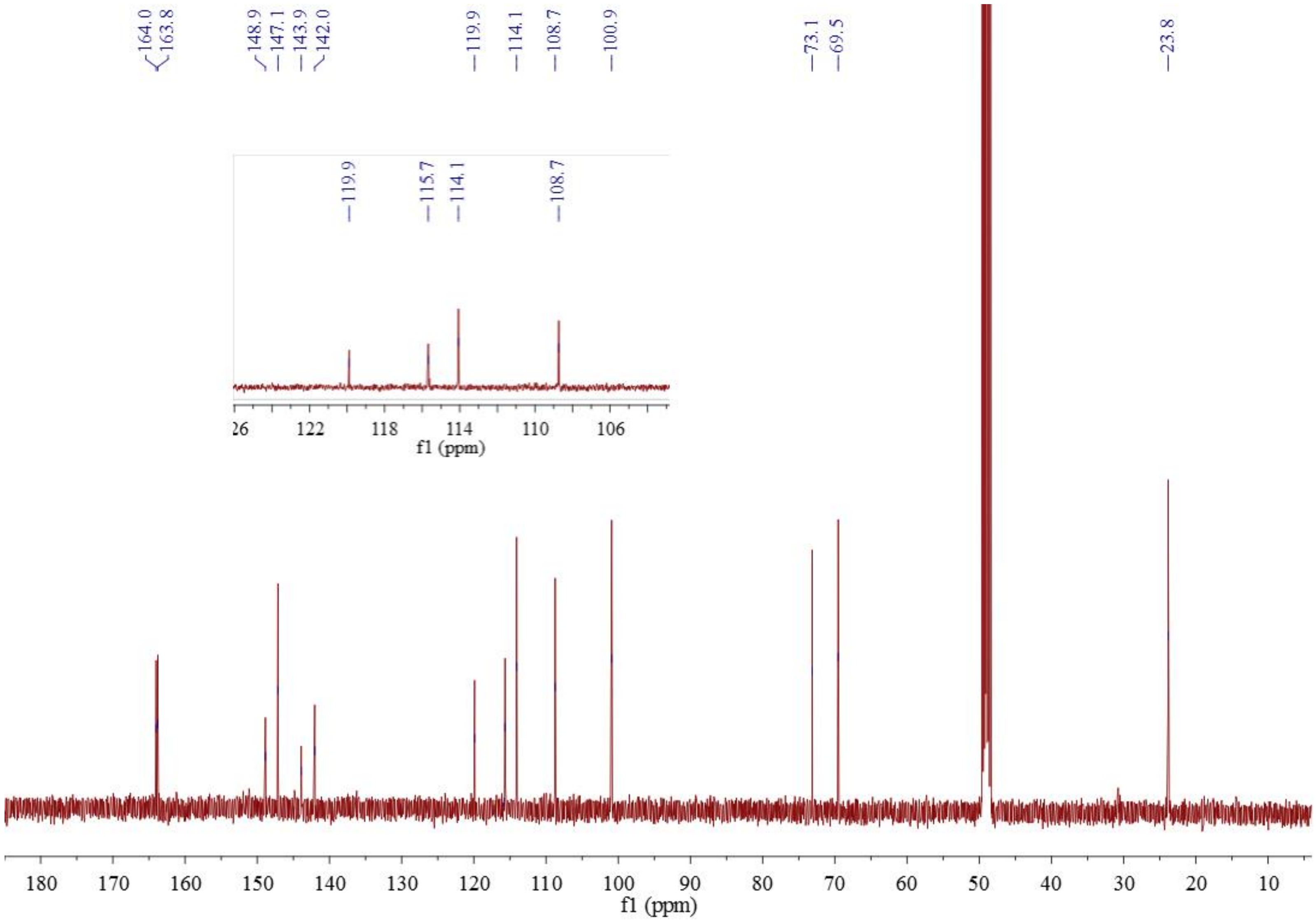

Figure S9: HSQC spectrum of compound **2**

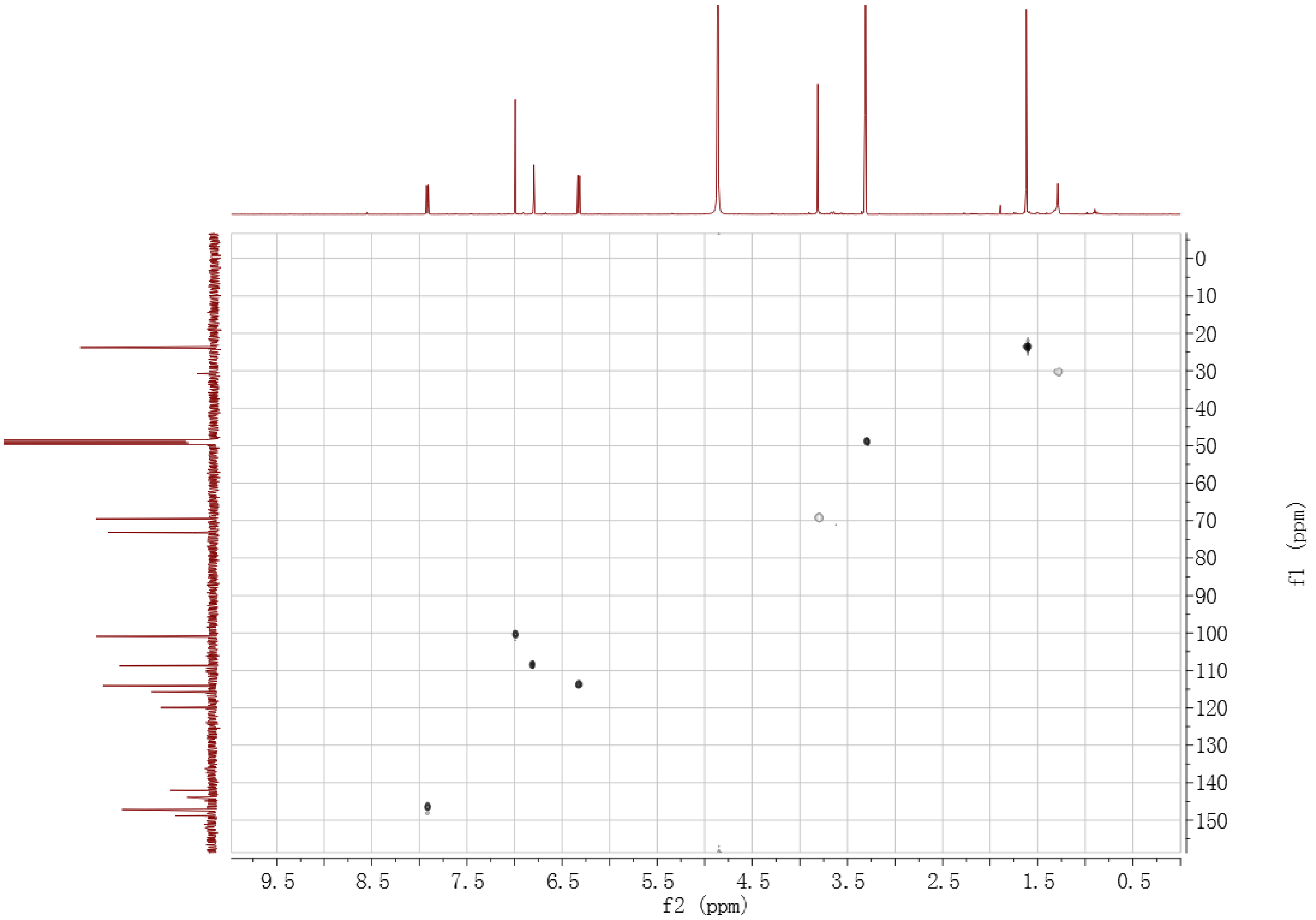

Figure S10: HMBC spectrum of compound **2**

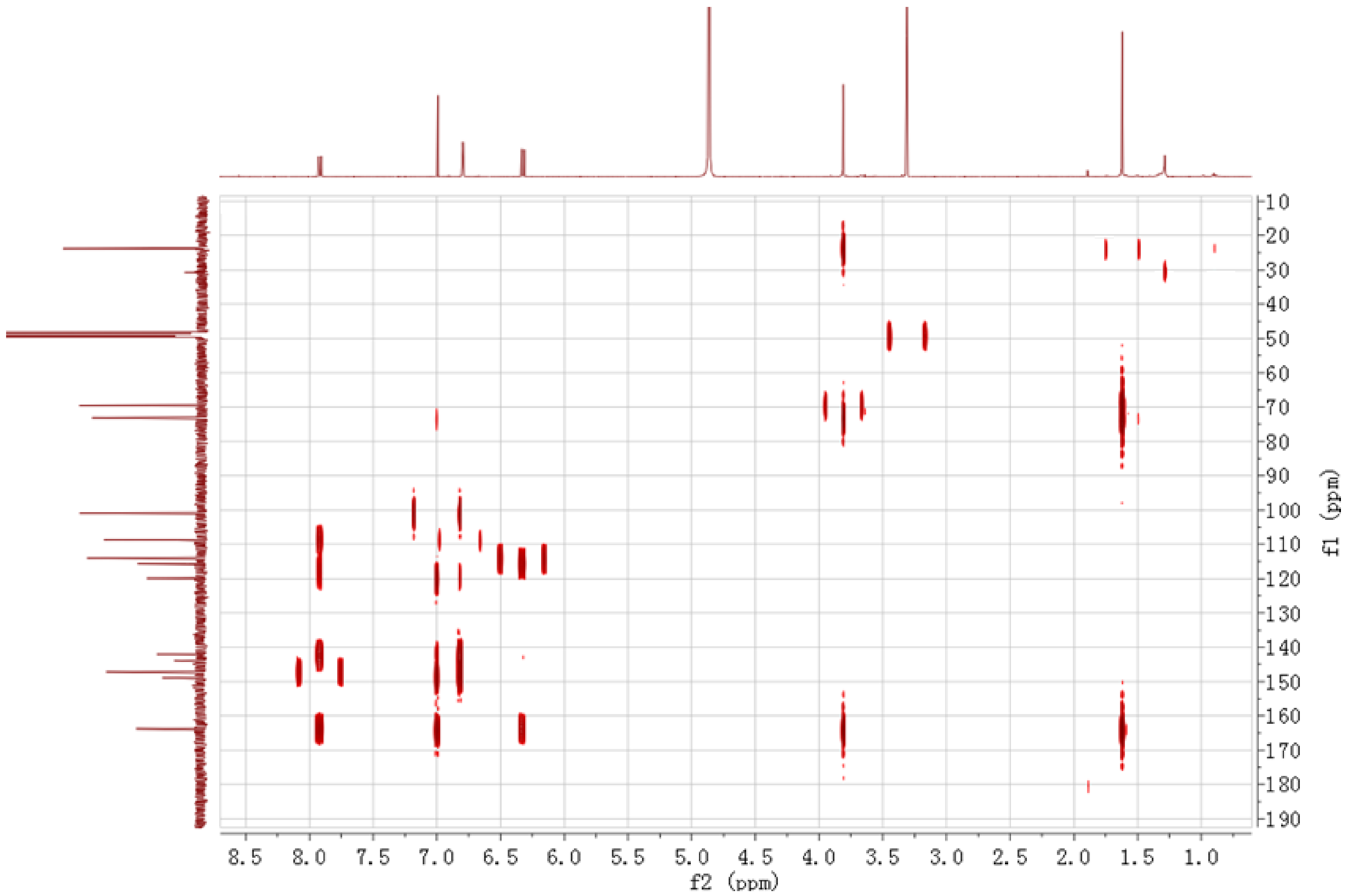

Figure S11: HR-ESI-MS(+) of compound **8**

Mass Spectrum SmartFormula Report

Analysis Info

Analysis Name	D:\Data\MS\data\201406\hulizhi-YLR-A2-13_pos.d	Acquisition Date	6/6/2014 8:40:24 AM
Method	POS_100-2000_Dirrect Infusion.m	Operator	BDAL@DE
Sample Name	SCSIO	Instrument / Ser#	maXis 29
Comment			

Acquisition Parameter

Source Type	ESI	Ion Polarity	Positive	Set Nebulizer	0.3 Bar
Focus	Not active	Set Capillary	4500 V	Set Dry Heater	180 °C
Scan Begin	100 m/z	Set End Plate Offset	-500 V	Set Dry Gas	4.0 l/min
Scan End	1500 m/z	Set Collision Cell RF	2000.0 Vpp	Set Divert Valve	Waste

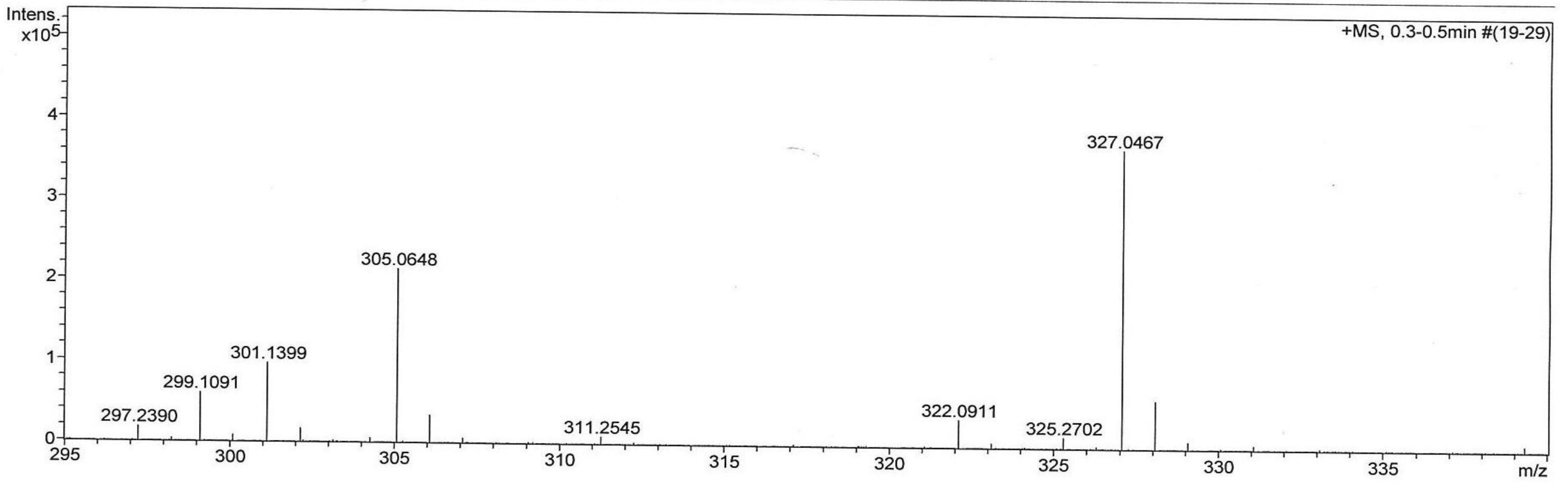

Meas. m/z	#	Formula	m/z	err [ppm]	Mean err [ppm]	rdb	N-Rule	e⁻ Conf	mSigma	Std I	Std Mean m/z	Std I VarNorm	Std m/z Diff	Std Comb Dev
305.0648	1	C 15 H 13 O 7	305.0656	2.7	2.8	9.5	ok	even	2.9	5.7	0.9	2.4	0.3	889.6
327.0467	1	C 15 H 12 Na O 7	327.0475	2.5	2.5	9.5	ok	even	1.5	3.3	0.8	1.6	0.2	884.3

Bruker Compass DataAnalysis 4.0 printed: 6/6/2014 8:47:40 AM Page 1 of 1

Figure S12: ^{1}H-NMR spectrum of compound **8**

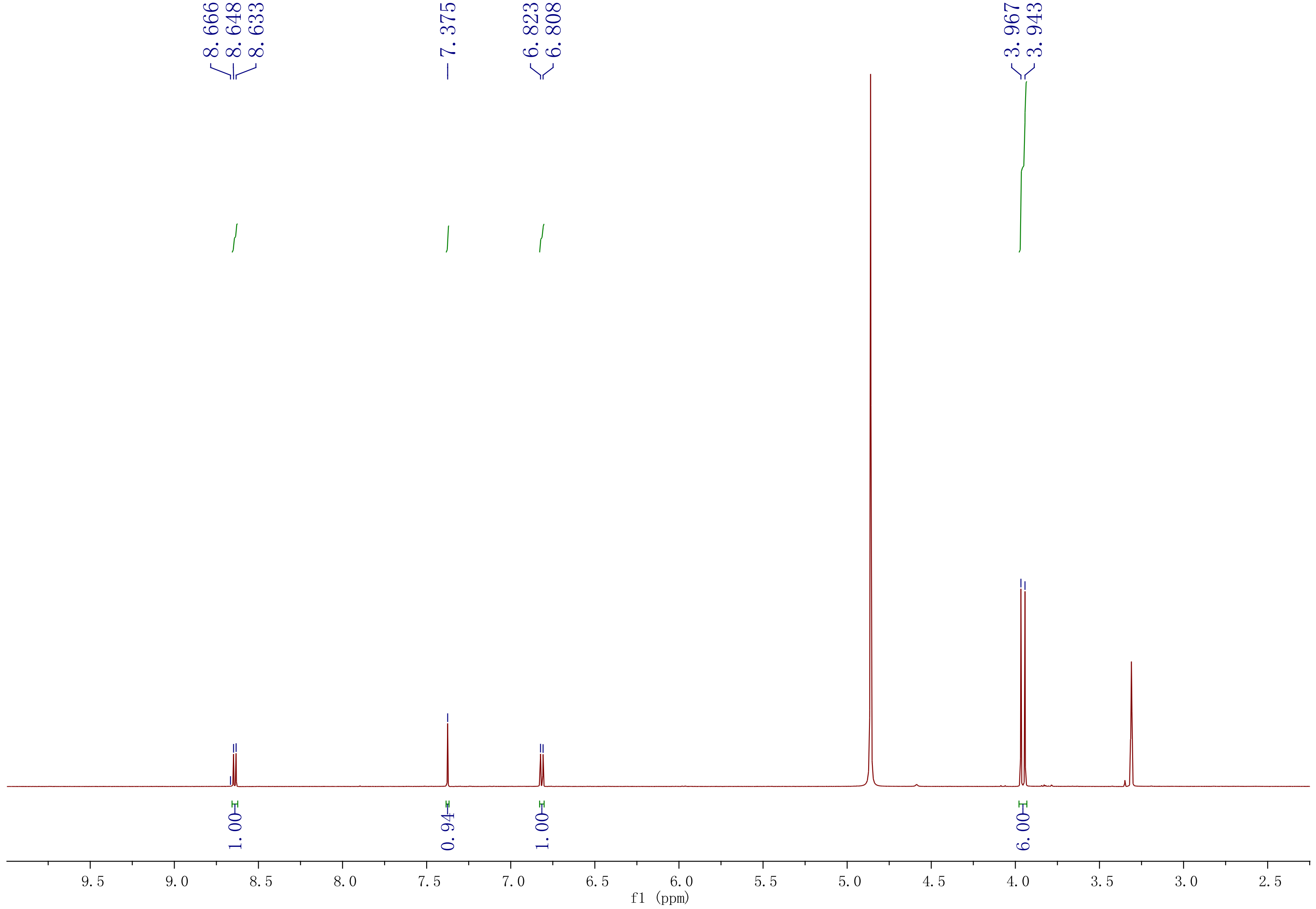

Figure S13: ^{13}C-NMR spectrum of compound **8**

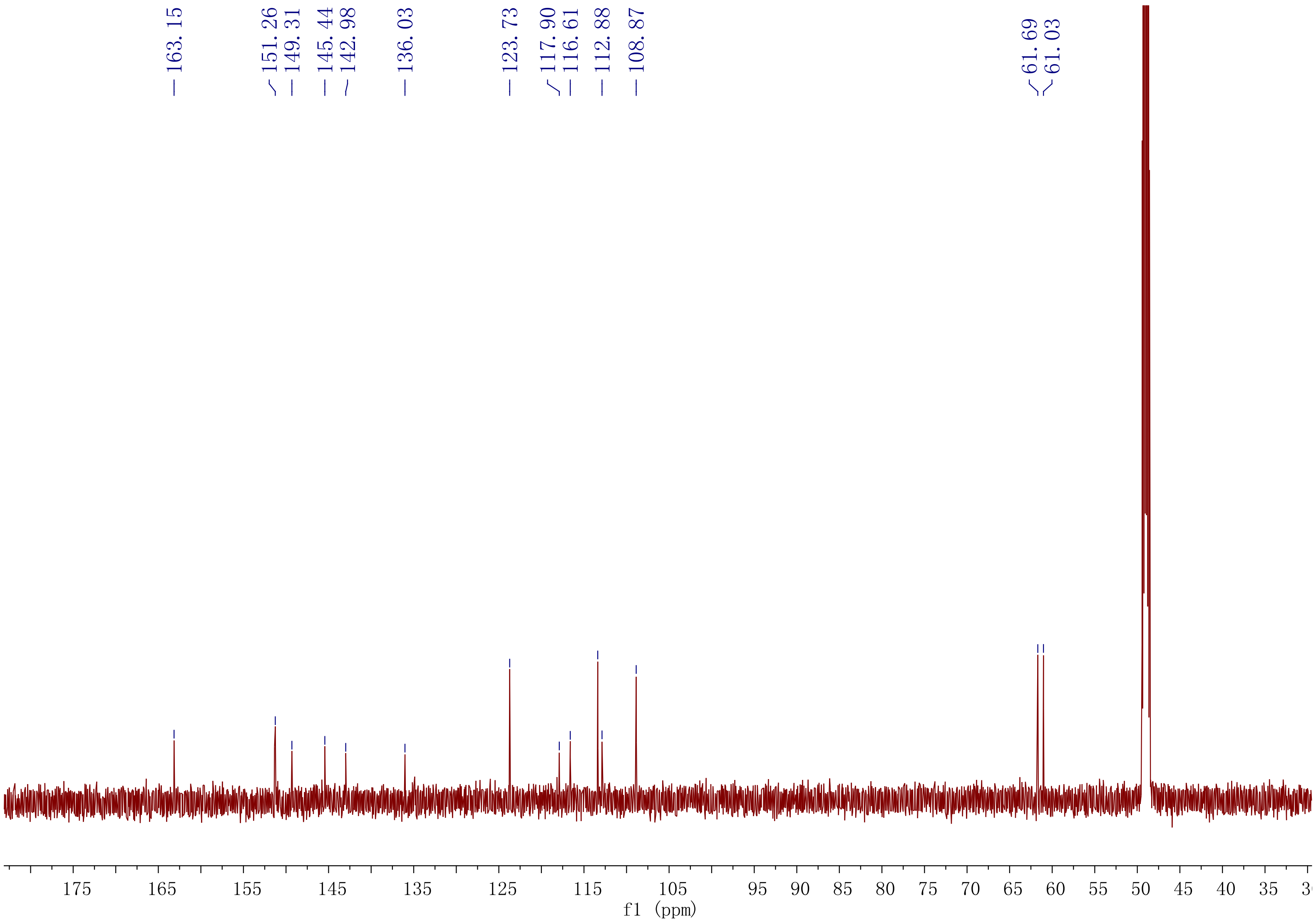

Figure S14: HSQC spectrum of compound **8**

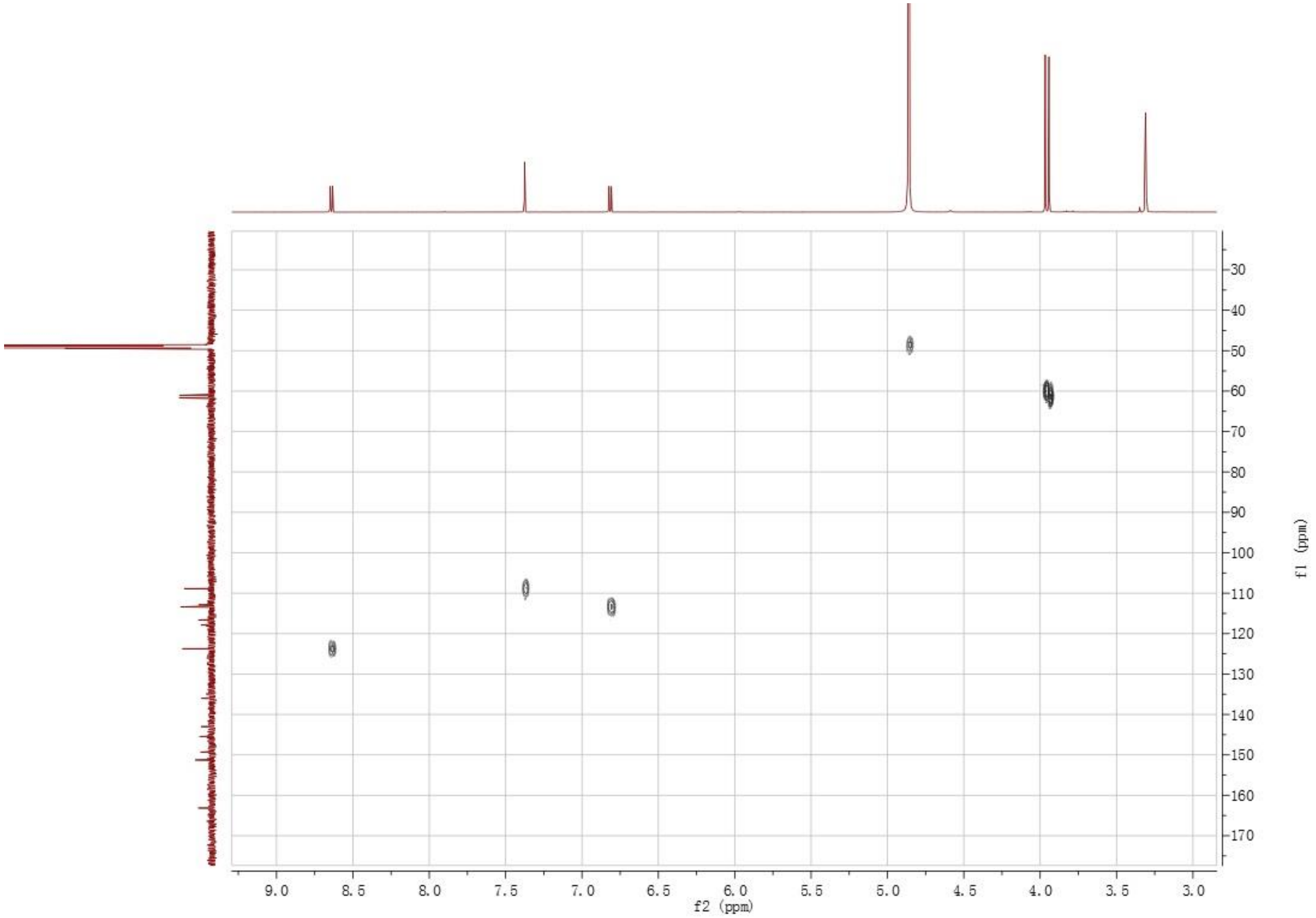

Figure S15: HMBC spectrum of compound **8**

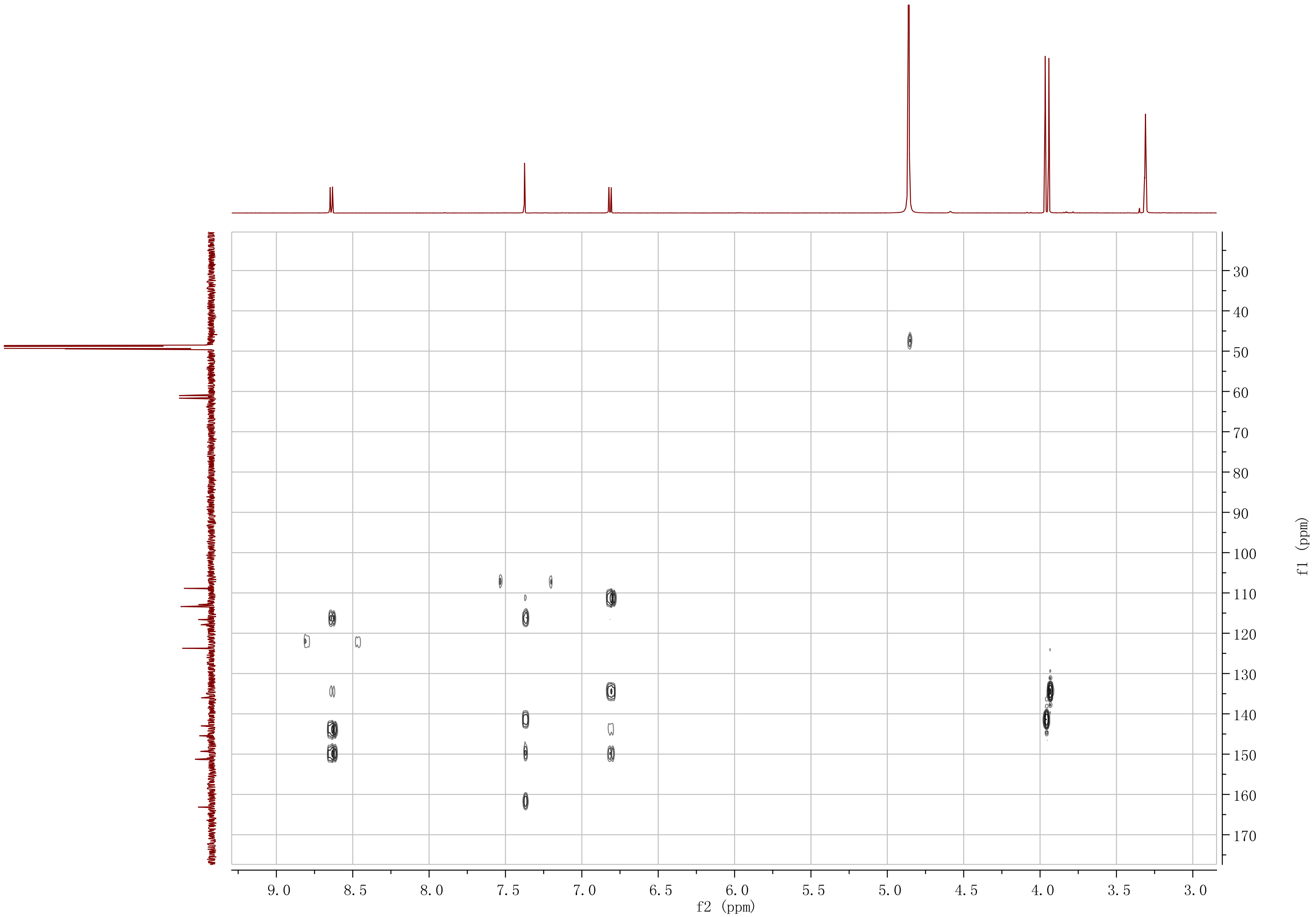

Supplement: Supplementary file 1 [file molecules-27-03334-s001.zip › molecules-1713636-supplementary.pdf]
